# The community-based prevention of diabetes (ComPoD) study: a randomised, waiting list controlled trial of a voluntary sector-led diabetes prevention programme

**DOI:** 10.1186/s12966-019-0877-3

**Published:** 2019-11-27

**Authors:** Jane R. Smith, Colin J. Greaves, Janice L. Thompson, Rod S. Taylor, Matthew Jones, Rosy Armstrong, Sarah Moorlock, Ann Griffin, Emma Solomon-Moore, Michele S. Y. Biddle, Lisa Price, Charles Abraham

**Affiliations:** 1 Institute of Health Research, University of Exeter Medical School, St Luke’s Campus, Heavitree Road, Exeter, EX1 2LU UK; 2 School of Sport, Exercise & Rehabilitation Sciences, University of Birmingham, Edgbaston, Birmingham, B15 2TT UK; 3 Medical Research Council/Chief Scientist Office Social & Public Health Sciences Unit, University of Glasgow, 200 Renfield Street, Glasgow, G2 3AX UK; 4 Department of Health & Social Sciences, University of the West of England, Frenchay Campus, Coldharbour Lane, Bristol, BS16 1QY UK; 5 Research Ethics & Governance Office, University of Exeter, Lafrowda House, St Germans Road, Exeter, EX4 6TL UK; 6 Cancer Sciences, University of Southampton, Southampton, SO17 1BJ UK; 7 Department for Health, University of Bath, Bath, BA2 7AY UK; 8 School of Sport & Health Sciences, University of Exeter, St Luke’s Campus, Heavitree Road, Exeter, EX1 2LU UK; 9 School of Psychological Sciences, Faculty of Medicine, Dentistry and Health Sciences, University of Melbourne, Melbourne, VIC 3010 Australia

**Keywords:** Randomised controlled trial, Diabetes prevention, Weight loss, Diet, Physical activity, Voluntary sector

## Abstract

**Objective:**

This two-site randomised trial compared the effectiveness of a voluntary sector-led, community-based diabetes prevention programme to a waiting-list control group at 6 months, and included an observational follow-up of the intervention arm to 12 months.

**Methods:**

Adults aged 18–75 years at increased risk of developing type 2 diabetes due to elevated blood glucose and being overweight were recruited from primary care practices at two UK sites, with data collected in participants’ homes or community venues. Participants were randomised using an online central allocation service. The intervention, comprising the prototype “Living Well, Taking Control” (LWTC) programme, involved four weekly two-hour group sessions held in local community venues to promote changes in diet and physical activity, plus planned follow-up contacts at two, three, six, nine and 12 months alongside 5 hours of additional activities/classes. Waiting list controls received usual care for 6 months before accessing the programme. The primary outcome was weight loss at 6 months. Secondary outcomes included glycated haemoglobin (HbA1c), blood pressure, physical activity, diet, health status and well-being. Only researchers conducting analyses were blinded.

**Results:**

The target sample of 314 participants (157 each arm) was largely representative of local populations, including 44% men, 26% from ethnic minorities and 33% living in deprived areas. Primary outcome data were available for 285 (91%) participants (141 intervention, 144 control). Between baseline and 6 months, intervention participants on average lost more weight than controls (− 1.7 kg, 95% CI − 2.59 to − 0.85). Higher attendance was associated with greater weight loss (− 3.0 kg, 95% CI − 4.5 to − 1.5). The prototype LWTC programme more than doubled the proportion of participants losing > 5% of their body weight (21% intervention vs. 8% control, OR 2.83, 95% CI 1.36 to 5.90) and improved self-reported dietary behaviour and health status. There were no impacts on HbA1c, blood pressure, physical activity and well-being at 6 months and, amongst intervention participants, few further changes from six to 12-months (e.g. average weight re-gain 0.36 kg, 95% CI − 0.20 to 0.91). There were no serious adverse events but four exercise-related injuries were reported in the intervention arm.

**Conclusions:**

This voluntary sector-led diabetes prevention programme reached a broad spectrum of the population and had modest effects on weight-related outcomes, but limited impacts on other diabetes risk factors.

**Trial registration:**

Trial registration number: ISRCTN70221670, 5 September 2014

Funder (National Institute for Health Research School for Public Health Research) project reference number: SPHR-EXE-PES-COM.

## Background

Diabetes and its complications account for around 12% of global health expenditure [1]. If recent trends continue, by 2040 over 600 million people worldwide will have diabetes [1]. Prevention of type 2 diabetes is paramount to curb this growing health crisis [2, 3].

Drawing on international evidence from high quality trials [4–6] and systematic reviews [7, 8], guidelines [3, 9, 10] recommend intensive lifestyle interventions promoting modest weight loss (e.g. 5% of body weight [9]) through changes in diet and physical activity to prevent or delay progression to diabetes in people at high risk of the condition. Without intervention, 30–50% of people with one or more markers of non-diabetic hyperglycaemia [11], a state of chronically raised blood glucose indicated by impaired fasting glucose, impaired glucose tolerance, or raised HbA1c (glycated haemoglobin of 42–47 mmol/mol) [3], will develop type 2 diabetes within 5 years [12]. Announced in March 2015, the “Healthier You: NHS Diabetes Prevention Programme” (NHS DPP) launched in 2016 to target the estimated 11% of people across England with non-diabetic hyperglycaemia [13], with more than 280,000 people referred as of September 2018 [14].

Despite roll-out of the NHS DPP, UK National Institute for Health and Care Excellence (NICE) diabetes prevention guidance [3] states that “evidence on both the short- and long-term effectiveness and cost-effectiveness of translating prevention trials into UK practice” (p.157) is still lacking. A systematic review [15] reported that “real-world” diabetes prevention interventions demonstrating greater adherence to recommendations on intervention content [3] generated higher levels of weight loss (up to 4 kg at 12 months for the most guideline-adherent). Preliminary data from the NHS DPP [14] indicate a mean weight loss of 3.2 kg amongst the half of participants who attended at least 60% of the sessions, which is sufficient to affect diabetes risk [3, 5]. Cautious interpretation is warranted, however, because this finding was based on a selective sub-group of participants, without reference to a comparison group. Moreover, no other UK community-based programmes [16–18] have demonstrated weight loss at this level in controlled studies with high levels of follow up.

Therefore, there remains a need for further, robust evidence on the implementation and effectiveness of pragmatic, guideline-based programmes in real-world UK communities that differ from the intensive lifestyle-based interventions studied in highly selected samples in the original diabetes prevention efficacy trials [4, 6, 19]. This paper reports a trial providing such evidence for a prototype version of “Living Well, Taking Control” (LWTC), a community-based diabetes prevention programme delivered by voluntary sector providers designed to be compliant with UK guidance [2, 3] that following in depth evaluation of retention and outcomes, as well as participant feedback, was subsequently adapted for delivery in the NHS DPP [20]. The voluntary sector is increasingly involved in delivering public health and health services in England [21] and has the potential to intervene at relatively low cost without employing over-stretched NHS staff, and to engage hard-to-reach communities [22]. These services are, however, rarely subject to robust evaluation [22].

This Community-based Prevention of Diabetes (ComPoD) trial aimed to assess whether the prototype LWTC programme was superior to usual care in promoting weight loss (primary outcome), modifying other risk factors (including HbA1c and physical activity) and improving self-reported outcomes in adults at risk of type 2 diabetes at 6 months of follow up. It also assessed whether any changes in outcomes amongst intervention participants were maintained up to 12 months, and to address concerns about the potential for such interventions to widen health inequalities [23], explored potential variations in effects across population subgroups.

## Methods

### Design

**Fig. 1:**
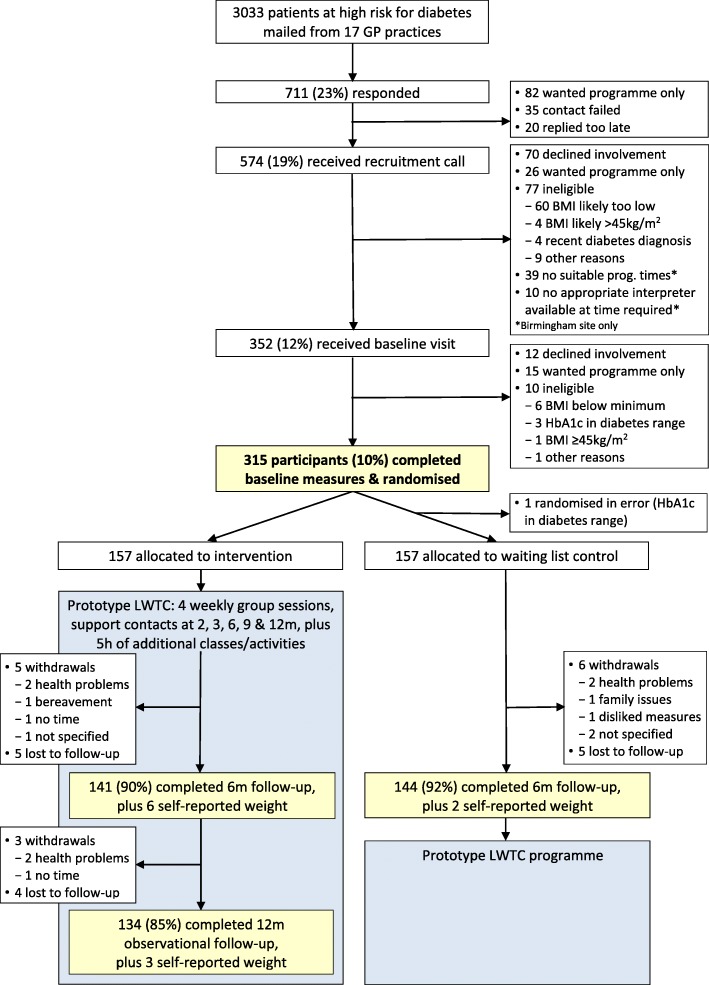
Recruitment and flow of participants through the ComPoD trial and prototype LWTC programme

We conducted a two-site, randomised, superiority trial with a parallel control arm, which is reported here in line with CONSORT guidelines (see Fig. 1 and Additional file 1: CONSORT checklist). Participants at two sites were individually allocated in a 1:1 ratio to the prototype LWTC programme (intervention), or a six-month, usual care waiting list for the programme (control). We used a waiting list control because the prototype LWTC programme was an existing service that eligible participants could access outside the trial, and it was deemed unethical and likely to jeopardise trial recruitment to deny access beyond 6 months. We also conducted a 12-month observational follow-up of intervention participants to assess maintenance of any changes beyond the core contact sessions.

### Participants

Trial inclusion criteria mirrored existing programme eligibility. From November 2014 to June 2015, general practitioner (GP) practices searched patient records to identify adults aged 18–75 years at high risk for type 2 diabetes due to elevated blood glucose (Fasting Plasma Glucose 6.1–6.9 mmol/l [24], or HbA1c 42–47 mmol/mol [3, 25] recorded within the past year) and body mass index (BMI) ≥25 kg/m^2^ (≥23 for South Asians [3]) and less than 45 kg/m^2^. Participants resided in or around Birmingham (the largest and most ethnically diverse English city outside London) or Exeter (a small city in rural south-west England).

We excluded participants who had a terminal illness, an existing diagnosis or baseline HbA1c (≥48 mmol/mol) indicating type 2 diabetes [25], were participating in another weight loss study, lacked capacity to give informed consent or understand study procedures despite assistance (e.g. due to dementia, severe learning disability), were pregnant, or could not be weighed using standard scales. The programme otherwise aimed to include and cater for people with low literacy, needing interpreters or with mild-moderate mental health problems, learning or physical disabilities.

### Procedures

The programme providers or Local Clinical Research Networks approached GP practices in eligible areas. Participating practices mailed potential participants an invitation providing brief information on the trial, a booklet on diabetes risk [26], a flyer on the prototype LWTC programme and a postage-paid reply slip. Trial researchers, with assistance from interpreters where requested, telephoned respondents to check likely eligibility, discuss participation and schedule an assessment. They then sent a full Participant Information Sheet and Consent Form. At an assessment visit in the participant’s home or another convenient location (e.g. university premises, GP practice), researchers answered any questions, obtained written informed consent and completed baseline measures, starting with BMI and HbA1c to confirm eligibility. People indicating that they wished to access the programme without participating in the trial were referred to the providers and added to their standard waiting list.

### Intervention

The voluntary sector providers (Westbank in Exeter www.westbank.org.uk, Health Exchange in Birmingham www.healthexchange.org.uk) had collaboratively designed the prototype LWTC programme, building on their previous well-being and diabetes management programmes [27]. Delivery was supported by a detailed intervention manual for programme facilitators and 40-page booklet summarising key information which participants used to document their clinical measures, goals, plans, and reflections. As documented in Additional file 2, these materials aimed to ensure that the programme structure, content (e.g. interactive information on healthy eating recommendations, aerobic and muscle-strengthening physical activity, impact of lifestyle on diabetes risk), behaviour change techniques used (e.g. motivational interviewing, goal setting, action planning, self-monitoring, engaging social support, problem-solving), and delivery (e.g. staff were trained in using person-centred counselling techniques to build empathy and assess and enhance motivation with a focus on building the perceived importance of, and confidence in, making changes) reflected all 11 NICE recommendations for diabetes prevention interventions [3]. Fidelity of implementation was checked at one site, and as reported elsewhere [28], found to be high. Participant satisfaction with the programme is also reported elsewhere [29].

The “core” intervention comprised four, weekly, two-hour sessions in groups of up to 12 participants (plus accompanying partners/supporters where desired) held in local community venues and led by trained facilitators. There were planned support contacts at two, three, six, nine and 12 months, which in Exeter were mostly group-based and in Birmingham mostly individual telephone calls. The programme was also designed to offer participants 5 hours of additional classes or activities of their choosing (e.g. exercise sessions, cooking classes). All available data on attendance at group sessions and follow up contacts were obtained from provider records at the end of the study, however, attendance at additional classes/activities had not been documented due to some of these taking place outside the provider organisations (e.g. walks in local communities) or being initiated by participants (e.g. visits to the provider’s gym).

### Control arm

Control participants went onto a six-month waiting list for the programme during which time they continued with routine care from their GP involving minimal or no follow-up related to their diabetes risk.

### Outcomes

Trained researchers, accompanied by an interpreter where necessary (*n* = 8), assessed outcomes at baseline and 6 months (mean 197 days) and, for intervention participants only, after 12 months (mean 375 days).

The primary outcome was weight loss (kg) from baseline to 6 months assessed using Tanita scales (model BC-601). Secondary weight-related outcomes were achievement of 3 and 5% weight loss, BMI (calculated from weight, and height measured using a Seca 213 portable stadiometer), and waist circumference (measured using a Seca measuring tape 201 placed between the uppermost border of the hip bone and the lower border of the rib cage against the skin, or light clothing). For these outcomes the average of two measurements, or three if the first two were > 0.5 units apart, was used. Other secondary objective outcomes were HbA1c, assessed using a portable Alere Afinion AS100 Analyzer to analyse capillary blood from a finger-prick, and blood pressure measured from the left arm whilst seated over bare skin or light clothing using an Omron 705IT monitor. Physical activity was assessed using Actigraph wGT3X accelerometers, which participants were asked to wear on their waist for seven consecutive days and were set to record activity counts in one-second epochs.

Participants also completed questionnaires routinely used in the LWTC programme as part of an existing before-after service evaluation [29]. These included the short-version New Zealand Physical Activity Questionnaire [30] (data not reported here due to availability of accelerometer data), a 27-item Fat and Fibre questionnaire [31] previously adapted for another diabetes prevention study [32] to assess dietary behaviours related to fat and fibre intake, and the Short Warwick-Edinburgh Mental Well-being Scale [33] to assess the frequency of positive thoughts and feelings over the past 2 weeks. Health status was assessed using the EuroQol EQ-5D [34] and life satisfaction with a widely-used single item 1–10 rating scale [35]. Participants who, when contacted for follow-up, wished to withdraw or were unavailable for face-to-face assessment, were asked for their self-reported weight. Socio-demographic (age, gender, ethnicity) and socio-economic characteristics (employment status, education level, Index of Multiple Deprivation derived from postcodes), co-morbidities, prescribed medications and co-interventions were recorded at baseline.

### Sample size

The standard deviation for weight loss in other UK community-based weight loss trials [36–38] is around 5.8 kg. To detect a minimum clinically important difference of 2.0 kg in weight loss [39] between arms, with 80% power and a two-sided alpha of 5%, we needed 133 per group. Allowing for 15% drop-out at 6 months based on our previous community-based weight loss studies [38, 40, 41], we sought to recruit 156 participants per arm.

### Randomisation and blinding

Participants were randomised at the end of their baseline assessment by researchers accessing an internet-based central allocation service developed and maintained by the Peninsula Clinical Trials Unit. Randomisation was stratified by site (Birmingham, Exeter) and a minimisation algorithm was used to balance the arms in terms of age (≤54, 55–64, 65–75 years), gender and baseline BMI (23–29.9, 30–36.9, 37–45 kg/m^2^), whilst maintaining a stochastic element [42]. Given the nature of the intervention and need for close liaison with the providers in relation to programme delivery, it was not possible to blind participants or researchers collecting data. The remainder of the research team, including those conducting analyses, remained blinded until primary analyses were complete.

### Analyses

A detailed statistical analysis plan was developed and agreed with the Trial Steering Committee including an independent statistician, prior to analysis. Entry of all primary outcome data and at least 20% of other data were checked by a second researcher.

Raw accelerometer data were initially downloaded using Actilife version 6.13.2 software then exported for processing in R-package GGIR (version 1.2–8) [43]. Data were analysed in one-second epochs with the first and final one-hour periods removed. A standard deviation of < 13 mg and an acceleration range < 50 mg on two axes were used to detect periods of non-wear, with non-wear periods calculated over 60 min using moving 15-min increments [44]. Time spent in moderate-to-vigorous-intensity physical activity overall, and as per guidelines at the time the study was conducted [45], accumulated in bouts of 10 min or more, was calculated using published Euclidean Norm Minus One (ENMO; mg) thresholds [46]. A valid day of measurement was defined as at least 600 min of registered wear-time [47]. Participants with a minimum of four valid days of activity including at least one weekend day were included in analyses, with data extrapolated where necessary to give estimates over 7 days.

Based on the intention-to-treat principle (i.e. analysis according to original random allocation), primary and secondary outcomes were compared between intervention and control arms at 6 months using linear (for continuous outcomes) or logistic (for binary outcomes) regression-based models in participants with complete primary outcome data. The primary analysis adjusted for baseline values of the outcome, stratification (site) and minimisation variables (gender, age and baseline BMI). Planned sensitivity analyses were also conducted with additional adjustment for ethnicity as a prognostically important variable that on visual inspection was identified as differing between arms at baseline.

Further sensitivity analyses examined the impact of imputing missing six-month primary outcome data (weight loss), using multiple imputation (assuming data missing at random), baseline value carried forward (commonly used in weight loss studies [36, 37]), and best and worst case scenarios (e.g. no change assumed for missing intervention cases and mean improvement seen in control completers assumed for missing control cases) [48]. For the primary outcome, we also conducted the completer analysis with self-reported weight data for the small number of participants unavailable for face-to-face measurement.

In further pre-specified analyses of the primary outcome, we explored the effects of programme attendance via a “per protocol” analysis where attendance at all four core group sessions was defined as a sufficient dose of the programme, and using a complier average causal effect (CACE) analysis [49, 50]. We examined the moderating effects of pre-specified baseline characteristics (site, gender, age, ethnicity, baseline BMI, deprivation index) on weight loss using regression models including an intervention-subgroup interaction term [51]. We also conducted an unplanned sensitivity analysis to examine the effect of excluding an extreme outlier.

All statistical tests were two-sided, and deemed statistically significant if *p* ≤ 0.05, with means and 95% confidence intervals (CI) reported. We made no adjustment for multiple testing because a primary outcome was pre-specified, and the outcomes are correlated, so simple adjustment for the number of comparisons would be overly conservative [52]. Data were analysed in STATA version 14.2.

### Service user involvement

The research built on prior work with service users [27] including workshops to understand the priorities and information needs of people at risk of diabetes [26]. Feedback from early participants in the prototype LWTC programme [29] confirmed prior to the trial that intervention burden and measures were acceptable. Meetings were held at the beginning and end of the study with a Public & Patient Involvement group comprising up to five previous programme participants. Two representatives of this group reviewed trial documentation in detail, sat on the Trial Steering Committee and assisted with interpretation of findings.

### Protocol amendments

During recruitment, inclusion criteria were modified to allow recruitment outside Exeter and to increase the upper age limit from 74 to 75 years to accommodate participants turning 75 between referral and baseline assessment. We also added collection of available laboratory-based HbA1c test results from GP practice databases for the year before and after baseline to allow comparison to lower than anticipated point-of-care test results.

Follow-up data on diagnoses (e.g. cancer) or co-interventions (e.g. new medications, participation in other weight loss programme) likely to affect weight, were ultimately only collected at one of the sites, precluding planned sensitivity analyses examining the effects of these factors on weight loss. Seven items intended to measure depression in the existing service evaluation [29] could not be scored due to lack of correspondence with the original, validated measure [53]. Finally, we adapted our original accelerometry analysis plans to accommodate recently developed processing methods for raw data [43]. Changes to the protocol and Statistical Analysis Plan were approved by the Trial Steering Committee.

## Results

### Participant flow (Fig. 1)

There was a 23% response rate to over 3000 invitations sent from 17 GP practices. Forty-four percent of respondents (314) were eligible, consented and randomised, representing 10% of those invited (2 to 26% across practices). A further 123 respondents (17%) opted for referral to the programme outside the trial.

Six-month primary outcome data were available for 285 (90% of intervention, 92% of control) participants. A further eight (3%) provided self-reported weight. At 12-months, 134 intervention participants (85%) provided objective weight data. Those not providing primary outcome data were similar to those providing data in terms of key socio-demographic, socio-economic and clinical characteristics except for being somewhat younger, and more likely in paid employment (Additional file 3: Table S1).

### Baseline characteristics

The randomisation produced balanced groups in terms of the key characteristics of gender, age and BMI categories overall (Table 1) and within each site (Additional file 3: Tables S2a, S2b). However, there were more White British participants and smokers in the intervention group, and intervention participants were on average 0.9 kg heavier at baseline. Since ethnicity was deemed to be potentially prognostic of weight loss [54], sensitivity analyses were conducted with adjustment for this as well as the planned adjustment for baseline weight in the primary analysis.

**Table 1:** *Participant characteristics at baseline (n (%) unless indicated)*

		Control *n* = 157 unless indicated	Intervention *n* = 157 unless indicated	Total sample *n* = 314 unless indicated
Stratification & minimisation variables				
Site	Exeter	86 (55)	84 (54)	170 (54)
	Birmingham	71 (45)	73 (47)	144 (46)
Gender (men)		69 (44)	68 (43)	137 (44)
Age categories	18–54 years	39 (25)	39 (25)	78 (25)
	55–64 years	43 (27)	45 (29)	88 (28)
	65–75 years	75 (48)	73 (47)	148 (47)
BMI categories	23–29.99 kg/m^2^=	65 (41)	64 (41)	129 (41)
	30–36.99 kg/m^2^	72 (46)	72 (46)	144 (46)
	37–45 kg/m^2^	20 (13)	21 (13)	41 (13)
Socio-demographic & socio-economic characteristics				
Mean (SD) age (years)		61.29 (9.86)	61.46 (9.99)	61.38 (9.91)
Ethnicity	White British	107 (68)	124 (79)	231 (74)
	White other	10 (6)	2 (1)	12 (4)
	Asian	26 (17)	20 (13)	46 (15)
	Black	12 (8)	10 (6)	22 (7)
	Mixed	2 (1)	1 (1)	3 (1)
Employment status	Retired	73 (47)	79 (50)	152 (48)
	Employed/self employed	65 (41)	56 (36)	121 (39)
	Unemployed	12 (8)	13 (8)	25 (8)
	Long-term sick/disabled	2 (1)	3 (2)	5 (2)
	Carer	1 (1)	4 (3)	5 (2)
	Other	4 (3)	2 (1)	6 (2)
Level of education	Primary	5 (3)^a^	2 (1)	7 (2)^b^
	Some secondary	5 (3)^a^	2 (1)	7 (2)^b^
	Secondary to 16 years	52 (33)^a^	50 (32)	102 (33)^b^
	Secondary to 18 years	10 (6)^a^	11 (7)	21 (7)^b^
	Additional training	47 (30)^a^	59 (38)	106 (34)^b^
	Undergraduate	24 (15)^a^	22 (14)	46 (15)^b^
Postgraduate		13 (8)^a^	11 (7)	24 (8)^b^
Median (IQR) Index of Multiple Deprivation score (IMD)		20.38 (21.33)	19.42 (21.72)	19.75* (21.19)
IMD quintiles	1 most deprived	16 (10)	21 (13)	37 (12)
	2	32 (20)	33 (21)	65 (21)
	3	35 (22)	39 (25)	74 (24)
	4	42 (27)	32 (20)	74 (24)
	5 least deprived	32 (20)	32 (20)	64 (20)
Clinical characteristics				
Any long term condition		114 (73)	115 (74)^c^	229 (73)^d^
Smoking		13 (8)	22^a^ (14)	35^b^ (11)
Disability		20 (13)	19 (12)	39 (12)
Mean (SD) weight (kg)		86.82 (13.37)	87.74 (16.76)	87.28 (15.14)
Mean (SD) HbA1c (mmol/mol) from point-of-care test		39.69 (2.73)	39.88 (3.06)	39.79 (2.90)
HbA1c in non-diabetic hyperglycaemia range (42–47 mmol/mol) from point-of-care test		42 (27)	45 (29)	87 (28)

*Compared to 17.4 for England [57]

^a^*n* = 156; ^b^*n* = 313; ^c^*n* = 155; ^d^*n* = 312

There was good representation of men (44%), ethnic minorities (26%) and people living in deprived areas (33% in the two most deprived quintiles) in the sample, which comprised mainly older (mean age 61.4 years), retired (48%), obese adults (mean BMI 31.7) with existing long-term conditions (73%), most commonly hypertension, high cholesterol and musculoskeletal problems. Only 17% met recommendations for 150 min per week of moderate-vigorous physical activity accumulated in bouts of at least 10 min.

The mean sample HbA1c (39.8 mmol/mol) according to trial point-of-care testing was below the 42–47 mmol/mol range for non-diabetic hyperglycaemia, with only 87 participants (28%) having a point-of-care HbA1c result in this range. However, 200 (92%) of 218 participants (69% of total sample) for whom laboratory-based HbA1c results were obtained from GP practices, had a record of HbA1c in the non-diabetic hyperglycaemia range during the prior year, with a mean (SD) of 43.9 (2.2).

The sample was representative of local populations, with half of Birmingham participants [55] and 7% of Exeter participants [56] from a non-White British background, and 58% overall (90% in Birmingham, 31% in Exeter) living in areas with above average levels of deprivation [57] (Additional file 3: Tables S2a, S2b). Compared to UK population norms, self-rated health (mean 73.9 on 0–100 scale) was somewhat lower (e.g. mean 81.7 for 55–64, 77.3 for 65–74 age groups [58]) and well-being similar (mean 24.9 on 0–35 scale compared to 23.6 for UK adults [33]).

### Intervention delivery and attendance

Attendance data were received from providers for 135 intervention participants (86%), of whom 129 (82%) had complete data on attendance at core group sessions (Additional file 3: Table S3). Attendance at these declined from 92% at session one to 81% at session four with 68% of participants attending all four group sessions (79% in Exeter, 56% in Birmingham), and an average of 3.4 sessions attended (3.7 in Exeter, 3.1 in Birmingham). Those attending all group sessions were significantly older, more likely to be White British, have lower education levels and be from less deprived areas, and less likely to be smokers than those missing sessions (Additional file 3: Table S4).

Data on follow up support contacts had not been consistently recorded by providers. Information on 2-, 3- and 6-month contacts, primarily delivered in groups in Exeter and via a mix of telephone (2 and 3 months) and groups (6 month) in Birmingham, were available for only 109 (69%), 91 (58%) and 53 (34%) participants, respectively. Data on contacts beyond 6 months were available for only six (4%) participants.

### Findings from primary analyses at six months

The main findings are presented in Table 2. The intervention group on average lost 1.7 kg more weight between baseline and 6 months than the waiting list control group (95% CI − 2.6 to − 0.9).

**Table 2:** Primary intention-to-treat analyses of primary and secondary outcomes at 6 months for participants providing data

	Waiting list control group		Intervention group		Between group difference*	*p*-value
	Baseline *n* = 157 unless indicated	6-months *n* = 144 unless indicated	Baseline *n* = 157 unless indicated	6-months *n* = 141 unless indicated	Mean (95% CI) or odds ratio (OR) (95% CI) where indicated	
Primary outcome						
Weight loss (kg)	n/a	−0.10 (3.03)	n/a	−1.91 (4.49)	−1.72 (−2.59 to − 0.85)	<0.001
Secondary weight-related outcomes						
N (%) > 3% weight loss	n/a	23 (16)	n/a	46 (33)	OR = 2.53 (1.41 to 4.52)	0.002
N (%) > 5% weight loss	n/a	12 (8)	n/a	29 (21)	OR = 2.83 (1.36 to 5.90)	0.006
Mean (SD) BMI (kg/m^2^)	31.56 (4.39)	31.58 (4.60)	31.85 (4.66)	31.07 (4.53)	−0.64 (−0.95 to − 0.33)	<0.001
Mean (SD) waist circumference (cm)	104.80 (11.28)	104.11 (11.48)	104.85 (12.62)	102.36 (12.50)	−1.36 (−2.36 to −0.36)	0.008
Other secondary objective outcomes						
Mean (SD) HbA1c (mmol/mol)	39.69 (2.73)	40.18^a^ (3.22)	39.88 (3.06)	39.71 (5.70)	−0.76 (−1.73 to 0.21)	0.124
Mean (SD) blood pressure systolic (mmHg)	137.58 (18.82)	138.44 (17.20)	136.22 (17.10)	136.91 (17.48)	−0.51 (−3.69 to 2.67)	0.752
Mean (SD) blood pressure diastolic (mmHg)	79.83 (10.60)	79.85 (9.33)	78.97 (10.37)	78.21 (9.77)	−0.81 (−2.62 to 1.01)	0.383
Mean (SD) total mins moderate-vigorous activity (MVPA) per week	318.67^b^ (176.07)	312.95^c^ (207.80)	337.34^d^ (189.12)	332.05^e^ (206.24)	−11.22 (−48.21 to 25.78)	0.551
Mean (SD) total mins MVPA in 10+ min bouts per week	73.55^b^ (96.67)	79.57^c^ (127.99)	80.09^d^ (116.85)	82.45^e^ (130.36)	−8.23 (−30.92 to 14.46)	0.475
N (%) meeting recommendation for 150 mins MVPA in 10+ min bouts per week	22^b^ (16)	20^c^ (19)	25^d^ (17)	20^e^ (18)	OR = 0.72 (0.31 to 1.68)	0.448
Secondary self-reported outcomes						
Dietary behaviour fat subscale scores (0 to 3, lower better)	1.95^f^ (0.39)	1.92^g^ (0.33)	1.94 (0.30)	1.81^h^ (0.33)	−0.11 (−0.18 to −0.05)	0.001
Dietary behaviour fibre subscale score (0 to 3, higher better)	2.17^f^ (0.40)	2.19^g^ (0.42)	2.17 (0.38)	2.30^h^ (0.38)	0.12 (0.05 to 0.20)	0.001
Mean (SD) health rating (0–100, higher better)	75.09^i^ (17.37)	73.65^j^ (18.49)	72.78^k^ (19.95)	77.24^h^ (16.66)	4.48 (0.85 to 8.11)	0.016
Mean (SD) life satisfaction rating (0–10, higher better)	7.43^i^ (1.90)	7.48^j^ (1.96)	7.65 (2.03)	7.73^h^ (1.74)	0.14 (−0.18 to 0.46)	0.385
Mean (SD) mental well-being scores (0–35, higher better)	24.88^f^ (5.09)	24.07^h^ (5.02)	24.82 (4.69)	24.99^h^ (3.89)	0.04 (−0.21 to 0.28)	0.761

*Mean differences, ORs and CIs adjusted for baseline outcome value, site, gender, age and baseline BMI category

^a^*n* = 142, 2 participants missing HbA1c due to machine malfunction;

^b^*n* = 135, ^c^*n* = 104, ^d^*n* = 143, ^e^*n* = 112, with 4+ valid days, including a weekend day, of accelerometry data

^f^*n* = 154; ^g^*n* = 141; ^h^*n* = 140; ^i^*n* = 152; ^j^*n* = 139; ^k^*n* = 156

The prototype LWTC programme doubled the proportion of participants achieving weight loss of 3 and 5% of their body weight, with 33% of intervention participants losing ≥3 and 21% losing ≥5%, compared to 16 and 8% of controls. There were significant differences between groups in terms of reductions in BMI and waist circumference, but not in HbA1c, blood pressure or objectively-measured moderate-vigorous physical activity.

In terms of self-reported outcomes, the intervention group showed significantly greater decreases in scores reflecting dietary behaviour related to fat intake and significantly greater increases in scores reflecting fibre intake, as well as significantly greater improvements in self-rated health status than controls. There were no significant differences in life satisfaction or mental well-being.

### Findings from sensitivity and subgroup analyses at six months

Findings in relation to the primary outcome were robust, ranging from − 1.5 to − 1.7 kg (CIs ranging from − 2.6 to − 0.8) greater weight loss at 6 months amongst participants in the intervention compared to control arm when various approaches were employed to impute missing weight data (Additional file 3: Table S5). When analyses of clinical outcomes were additionally adjusted for baseline between-group differences in ethnicity (white British versus other) most differences were slightly increased (e.g. adjusted mean difference in weight loss = − 1.9 kg, CI − 2.8 to − 1.0; Additional file 3: Table S6). Excluding an extreme outlier who gained 20 kg in the intervention group similarly increased between-group differences in weight loss (mean difference − 1.9 kg, CI − 2.7 to − 1.1).

Differences in weight loss were greater in per protocol analyses confined to intervention participants (*n* = 81, 57% of those with primary outcome data at 6 months) attending all four group sessions (mean difference to controls − 2.2 kg, CI − 3.1 to − 1.3) and in the CACE analysis (mean difference − 3.0 kg, CI − 4.5 to − 1.5).

Moderator analyses (Table 3) suggested effects of the programme on weight loss were similar across sites and regardless of gender or area deprivation. Though no between-subgroup differences were significant, those aged under 55 years and with BMI < 30 kg/m^2^ showed lower average weight loss (< 1 kg), and white British participants lost more weight than other ethnicities.

**Table 3:** Moderating effects of key baseline characteristics on weight loss (primary outcome) at 6 months (*n* = 285)^a^

	Between group difference^b^ Mean (95% CI)	Interaction term *p* value
Site		0.829
Exeter (*n* = 151)	−1.77 (−3.07 to − 0.46)	
Birmingham (*n* = 134)	−1.59 (−2.71 to − 0.48)	
Gender		0.508
Male (*n* = 124)	−1.87 (−3.43 to −0.31)	
Female (*n* = 161)	−1.43 (−2.40 to −0.45)	
Age (yrs)		0.501
<55 (*n* = 66)	−0.75 (−2.92 to 1.42)	
55–65 (*n* = 83)	−1.87 (−3.39 to − 0.36)	
65–75 (*n* = 136)	−2.16 (− 3.30 to − 1.02)	
Baseline BMI (kg/m^2^)		0.101
23.0–29.9 (*n* = 118)	−0.79 (− 1.91 to 0.34)	
30.0–36.9 (*n* = 130)	−1.99 (−3.36 to − 0.63)	
37.0–45.0 (*n* = 37)	−2.67 (−6.07 to 0.72)	
Ethnicity		0.562
White British (*n* = 211)	−2.03 (−3.09 to −0.96)	
Other (*n* = 74)	−1.10 (−2.65 to 0.45)	
Deprivation		0.643
Low (≤ England median IMD score) (*n* = 116)	−1.97 (−3.29 to −0.65)	
High (> England median IMD score) (*n* = 169)	−1.52 (−2.71 to −0.33)	

^a^based on intention-to-treat completers

^b^Adjusted for baseline outcome value, site, age, gender and baseline BMI

### Findings at 12 months (Table 4)

There was a small, non-significant average weight re-gain of 0.4 kg (CI − 0.2 to 0.9) amongst intervention participants between 6 and 12 months, representing an average weight loss of − 1.6 kg (CI − 2.5 to − 0.7) from baseline to 12 months in the intervention arm, but with only small numbers (6%) achieving 5% weight loss at 12 months. There was a small further reduction in waist circumference but no significant changes in other objective or self-reported outcomes.

**Table 4:** Maintenance of changes between 6- and 12-months amongst intervention participants with data at both timepoints

	6 months *n* = 134 unless indicated	12 months *n* = 134 unless indicated	Mean change (95% CI)	*p* value
Primary outcome				
Mean (SD) weight (kg)	85.52 (16.54)	85.88 (17.01)	0.36 (−0.20 to 0.91)	0.206
Secondary weight-related outcomes				
N (%) > 3% weight loss from baseline	45 (34)	15 (11)	N/A	
N (%) > 5% weight loss from baseline	28 (21)	8 (6)	N/A	
Mean (SD) BMI (kg/m^2^)	31.16 (4.60)	31.28 (4.78)	0.13 (−0.08 to 0.33)	0.239
Mean (SD) waist circumference (cm)	102.46^a^ (12.67)	101.96^a^ (12.59)	−0.64 (−1.29 to 0.00)	0.051
Other secondary objective outcomes				
Mean (SD) HbA1c (mmol/mol)	39.32^a^ (2.65)	39.52^a^ (3.09)	0.20 (−0.30 to 0.69)	0.438
Mean (SD) BP systolic (mmHg)	136.95^a^ (17.11)	137.91^a^ (19.28)	0.96 (−1.54 to 3.47)	0.448
Mean (SD) BP diastolic (mmHg)	77.90^a^ (9.83)	78.30^a^ (11.25)	0.40 (−1.02 to 1.82)	0.579
Mean (SD) total mins moderate-vigorous activity (MVPA) per week	344.87^b^ (221.54)	355.06^b^ (247.65)	10.18 (−23.01 to 43.39)	0.544
Mean (SD) total mins MVPA in 10+ min bouts per week	92.88^b^ (140.75)	84.78^b^ (137.82)	−8.09 (−28.34 to 12.15)	0.429
N (%) meeting recommendation for 150 mins MVPA in 10+ min bouts per week	17^b^ (20)	13^b^ (15)	NA	
Secondary self-reported outcomes				
Dietary behaviour fat subscale scores (0 to 3, lower better)	1.81^c^ (0.34)	1.83^c^ (0.31)	0.02 (−0.02 to 0.07)	0.362
Dietary behaviour fibre subscale score (0 to 3, higher better)	2.30^c^ (0.37)	2.28^c^ (0.39)	−0.03 (− 0.08 to 0.02)	0.293
Mean (SD) health rating (0–100, higher better)	77.34^c^ (16.71)	78.34^c^ (16.74)	1.00 (−1.74 to 3.74)	0.472
Mean (SD) life satisfaction rating (0–10, higher better))	7.74^b^ (1.75)	7.84^b^ (1.58)	0.10 (−0.15 to 0.34)	0.425
Mean (SD) mental well-being scores (0–35, higher better)	27.26^d^ (4.91)	27.57^d^ (4.57)	0.32 (−0.34 to 0.98)	0.342

^a^*n* = 133; ^b^*n* = 87; ^c^*n* = 130; ^d^*n* = 129

### Adverse events

No serious adverse events were deemed to be related to trial participation in either group. There were four non-serious adverse events amongst intervention group participants potentially related to the intervention, all short-term injuries stemming from increased exercise (pelvic pain, lower back pain, aggravation of existing sciatica, shoulder injury) that resolved on their own or with treatment (e.g. physiotherapy, acupuncture). There were none reported in the control group.

## Discussion

### Principal findings

Our results showed that the prototype LWTC voluntary sector-led diabetes prevention programme successfully engaged both men and women from a diverse and representative sample of the population. In this real-world setting, the programme generated an average 1.7 kg weight loss at 6 months compared to usual care. It also doubled the proportion of people losing ≥5% of their baseline weight, though only 21% of intervention participants achieved this level of weight loss. In sensitivity analyses, estimated effects on weight loss were robust to missing data (ranging from 1.5–1.7 kg) and increased when baseline differences, an extreme outlier and group session attendance were considered (up to 3 kg in CACE analysis). In observational follow-up of intervention participants, effects on weight were maintained to some degree at 12 months. The programme had no significant effects on other clinical risk factors (HbA1c and blood pressure), or moderate-to-vigorous physical activity, but resulted in changes in self-reported health status and dietary behaviours.

### Findings in relation to other studies

The prototype LWTC programme recruited a high proportion of men (44%) compared with previous studies of weight loss in UK settings (which have recruited between 16 and 31% males [36, 37, 59]). The magnitude of weight loss observed was similar to the 1.6 to 2.5 kg reported in reviews of randomised controlled trials of real-world diabetes prevention interventions [15] and previous UK-based diabetes prevention studies [16, 17], though less than the 3-4 kg that might be expected for programmes fully implementing NICE guidance on the content of diabetes prevention interventions [15]. Reasons for this may include lower than planned contact time (data on contacts outside core group sessions were not consistently recorded), or variations from planned delivery (fidelity was checked only in a sub-sample at one site [28]).

Since the weight change in our control group was close to zero and trial eligibility criteria and sample characteristics were almost identical to those of the NHS DPP, our data provide some confidence that the clinically meaningful weight loss (3.2 kg) observed for participants with high levels of attendance at NHS DPP sessions [14] is an accurate estimate. This estimate is also broadly consistent with our CACE analysis, which estimated a 3 kg weight loss for people attending all four core programme sessions.

The lack of effects on physical activity is a particular concern, given that a substantial proportion of programme time was devoted to promoting activity. Methods for enhancing physical activity in the prototype LWTC and similar programmes therefore need to be examined and, if necessary, refined in the light of what has been successful in other studies [18].

### Strengths and weaknesses

This is the first trial of a voluntary sector-led diabetes prevention intervention in the UK. Strengths included the fully-powered randomised controlled trial design, real-world setting, use of objective and independently assessed outcomes, high follow-up rates and representativeness of the study sample, including a high proportion of men, people from ethnic minority groups, and living in deprived areas. The findings are therefore likely to be reflective of programme performance across a range of UK locations.

Limitations included the 10% recruitment rate (which though it reduces the generalisability of findings is not atypical for such trials), the short (6 month) follow-up for comparative analyses due to ethical and practical constraints on the design, lack of blinding of data collectors (though objective measures were used) and incomplete attendance data. The wide variability in some effect estimates and a lack of power means that we cannot exclude the potential for effects on secondary outcomes (e.g. the sample size provided 80% power to detect changes of 66 min/week in MVPA and 1.4 mmol/mol in HbA1c), and differences in weight loss between participant sub-groups. Given the 7-month recruitment period, there is potential for seasonal effects on outcomes in both groups, however, this would not affect the main between-group comparison. It should also be noted that the prototype LWTC programme evaluated here has been substantially upgraded since the study, partly in response to our findings and to meet NHS DPP requirements [20], including expanding the number of initial sessions from four to seven, increasing the total number of formal contacts to 13 and placing greater emphasis on physical activity (e.g. mapping and discussing local opportunities to engage in ongoing physical activity, supporting enrolment in, and the development of new, walking groups). Hence, the programme’s performance may have been improved and ongoing evaluation is underway [14].

### Explanations and implications for clinicians and policymakers

The impact of individual-level health promotion interventions on health inequalities is often raised as a key public health policy question [23]. This study has shown that it is possible, through a voluntary sector delivery model, to recruit and engage both men and women with above average (for England) area deprivation scores and from a range of ethnic backgrounds. Moderation analyses showed that the effects of the prototype LWTC programme at 6 months were not significantly different across sites or population subgroups and extended to men, older adults, those with BMI in the obese range and those living in areas of above average deprivation. Although, we were unable to assess cost-effectiveness, a Social Return on Investment Analysis conducted as part of the wider evaluation of the programme estimated the cost at £296.95 per participant and that for every £1 invested, there was a social return of around £5.80 over a 3 year period [60].

An unanticipated finding was that based on point-of-care testing only 28% of participants had HbA1c in the non-diabetic hyperglycaemia range at baseline, despite available GP records confirming that over 90% had recent laboratory-tested HbA1c in the eligible range on referral. The mismatch could be explained by a tendency for point-of-care testing machines (which are not diagnostic tools [61]) to under-estimate HbA1c. Alternatively, there could be “diagnostic drift” between laboratory measures and baseline testing. In either case, this is an important participation-selection issue for large-scale diabetes prevention programmes. Previous research has also raised concerns about inconsistency between different methods for identifying people who are at risk of developing diabetes [62]. One approach to improving selection would be to require a second, confirmatory test (using the same method) and/or a higher cut-off for eligibility, to ensure that people entering the programme are truly “high risk”.

### Unanswered questions and future research

Further research is needed to establish robust, pragmatic criteria for identifying people at risk of type 2 diabetes, and to establish cost-effective methods for increasing physical activity and sustaining weight loss in real-world diabetes prevention programmes. We also urgently need research on alternative options for the high proportion of people who do not respond to invitations (up to 90%), choose not to attend (52%), or do not adhere sufficiently (around 50%) to available lifestyle interventions. Furthermore, work is needed to assess the in-service effectiveness of the upgraded LWTC programme and to identify and address any differential effects of current diabetes prevention programmes across population subgroups to minimise health inequalities.

## Conclusion

This study contributes to the limited base of robust evidence available on the “real-world” effectiveness of community-based diabetes prevention interventions in the UK, particularly voluntary sector-led programmes. Overall, our results confirm claims that voluntary sector-led programmes can reach a wide and diverse range of the population and show that the prototype LWTC programme had modest short-term effects on weight-related outcomes, including similar effects across population sub-groups who often fail to engage with other programmes. However, it had limited effects on other diabetes risk factors including physical activity. To maximise the impact on future diabetes incidence, similar diabetes prevention programmes may need to refine eligibility-testing procedures to appropriately target individuals at highest risk of diabetes, improve methods for supporting physical activity, and find ways to maximise programme attendance/contact time (which mediates weight loss) and the maintenance of any lifestyle changes generated.

## Supplementary information

**Additional file 1.** CONSORT checklist.

**Additional file 2.** Description of the prototype Living Well, Taking Control (LWTC) Programme.

**Additional file 3.** Supplementary tables.

## Data Availability

The dataset generated and analysed during the study will be made available through the University of Exeter’s Institutional Repository, Open Research Exeter (see https://ore.exeter.ac.uk). Access to these data is permitted but controlled through reasonable requests made via the repository to the chief investigator (Dr Jane Smith: jane.smith@exeter.ac.uk). Although use is permitted, this will be on the basis that the source of the data is acknowledged (including the funder) and it includes reference to the data set ‘handle’.

## Abbreviations

BMI: Body mass index
CACE analysis: complier average causal effect analysis
CI: Confidence intervals
ComPoD trial: Community-based Prevention of Diabetes trial
GP: General practitioner
HbA1c: Hemoglobin A1c (glycated haemoglobin)
IQR: Inter-quartile range
LWTC programme: Living Well, Taking Control programme
NHS DPP: National Health Service England Diabetes Prevention Programme
NICE: UK National Institute for Health and Care Excellence
SD: Standard deviation
