# Supplementary material for: The community-based prevention of diabetes (ComPoD) study: a randomised, waiting list controlled trial of a voluntary sector-led diabetes prevention programme
Source: Int J Behav Nutr Phys Act. 2019 Nov 27;16:112. doi: 10.1186/s12966-019-0877-3 (PMC6880578; doi:10.1186/s12966-019-0877-3)
Supplement: Supplementary file 2 — Additional file 2. Description of the prototype Living Well, Taking Control (LWTC) Programme. [file 12966_2019_877_MOESM2_ESM.docx]

**Supplementary file 1 – Description of the prototype Living Well, Taking Control (LWTC) Programme**

***Summary of NICE recommendations^s1^ on lifestyle interventions for diabetes prevention and how these mapped onto the structure, content and delivery of the prototype LWTC programme***

1. **Aim to promote changes in both diet and physical activity**

In line with evidence that the effectiveness of interventions for those at risk of diabetes is increased by targeting both diet and physical activity ^s2^, the aims of the prototype LWTC programme were to increase physical activity and improve diet to promote weight loss and enhance well-being. Four core semi-structured group education sessions covered: “Pre-diabetes & healthy lifestyle”, “Healthy eating”, “Physical activity” and “Positive mental health & well-being”. To further promote and maintain changes in diet and physical activity, follow-up access was provided according to individual goals and needs to relevant local activities and services (e.g. walking groups, other exercise classes, cooking classes, relaxation courses).

1. **Maximize frequency or number of contacts, within the resources available**

Use of trained facilitators to deliver core components, introducing participants to existing services and activities, and selected follow-ups in either a group setting or via phone allowed contact time and frequency to be maximised within the original Big Lottery funding for the programme, which was supplemented with the providers’ own funds to support completion of the trial.

1. **Provide at least 16 hours of contact time**

It was intended that participants would receive four two-hour core group education sessions (eight hours total), a 30 minute one-to-one session, five 30-minute follow-up contacts (three hours total), and access to at least five hours of classes or support for engagement in individually-tailored activities through existing services (see point 1). There were differences across the sites in how this was achieved: in Exeter there was often an initial one-to-one session and shorter first group session which each lasted an hour and follow-up contacts (especially at 6 months) were frequently delivered in groups; in Birmingham follow-up contacts were mainly undertaken on an individual basis via telephone (see Supplementary Table 3).

1. **Allow time between sessions, spreading them over a period of 9-18 months**

The group sessions were delivered weekly during the first month and follow-ups were scheduled at 2, 3, 6, 9 and 12 months. This allowed time between contacts for participants to make changes, review progress and goals, learn from experience, practice the self-regulatory skills taught in the sessions (see point 11) and identify and access any additional support required.

1. **Use a group size of 10-15 people**

The programme aimed to deliver to groups of up to 12 people.

1. **Ensure programmes adopt a person-centred, empathy-building approach**

The programme was designed to be participant-centred and individualised to participants’ needs. Motivational interviewing and patient-centred counselling techniques were used to build empathy ^s3^. These have been shown to be more effective than traditional didactic approaches to promoting behaviour change^s1^.

1. **Use established, well-defined behaviour change techniques**

Approaches used in the prototype LWTC programme were mapped against a list of key recommended techniques (points 8-11) that are associated with increased effectiveness in interventions to promote changes in diet and physical activity^s2^. These techniques were built into session plans to ensure that they were used repeatedly across core sessions. Additional techniques (e.g. reducing negative emotions, instruction on how to perform a behaviour, behavioural substitution) were used in specific sessions, and in the follow-up activities and services from which participants chose (e.g. demonstration of the behaviour and behavioural practice/rehearsal were present in exercise and cooking classes). All group facilitators received brief training in the use of these techniques prior to commencement of the trial. These techniques targeted factors identified by various social-cognitive models (e.g. Health Action Process Approach^s4^) as being important in motivating people to change their behaviour (e.g. risk perceptions, pros and cons of behaviour change, self-efficacy), translating motivations into action, and supporting longer-term maintenance of behaviour change^s2^ (e.g. self-regulatory approaches derived from theories such as Social Cognitive Theory^s5^ and Control Theory^s6^). The programme also adopted motivational approaches from self-determination theory^s7^, such as supporting intrinsic motivation, engaging social support/encouraging connectedness and building competence.

1. **Engage social support**

In line with evidence^s8^ that this increases intervention effectiveness, the prototype LWTC programme promoted engagement of support from participants’ family and friends in planning and making lifestyle changes, including bringing partners or other supporters along to sessions if desired. It also provided ample opportunities to access practical and emotional social support from peers within the programme.

1. **Provide information to raise awareness of the benefits of and types of lifestyle changes needed**

The core sessions provided information on, and addressed common misconceptions around, risk of diabetes, clinical risk indicators (e.g. HbA1c) and lifestyle changes to reduce risk. This was supported by educational materials about healthy eating (based on Food Standards Agency/Department of Health recommendations) and physical activity (based on recommendations for both moderate intensity aerobic activity and strength/resistance training^s9^). To accompany this and further enhance participants’ motivation to change, group facilitators received training in behaviour change techniques such as providing information about health consequences, weighing up pros and cons, comparative imagining of future outcomes and identifying discrepancies between current behaviour and goals.

1. **Explore and reinforce Importance and Confidence with graded goal-setting to build confidence**

All group facilitators received training on assessing, exploring and enhancing participants’ perceptions around the importance of making realistic changes to their diet and physical activity and their confidence to do so^s10^. Relevant behaviour change techniques to build confidence included the use of graded tasks within goal setting, problem-solving and the use of motivational interviewing techniques to explore and enhance confidence for behaviour change^,s3,s11^.

1. **Build on a coherent set of ‘self-regulatory’ intervention techniques**

Specific, individualised goal setting and action planning, use of self-monitoring tools (e.g. diaries, self-weighing), reviewing and providing feedback on performance of behaviours and outcomes (e.g. weight), planning for and addressing problems, and reviewing progress and goals^s11^ were central to the structure of the core sessions and follow-up contacts. Use of these self-regulatory approaches supported “learning from experience”.

**Underpinning facilitator training /competencies:** The prototype programme was delivered by in-house staff at the provider organisations who had professional qualifications including, for example, an undergraduate degree in nutrition or Register of Exercise Professionals (REPS) levels 4 & 3 certification. Facilitators had been recruited based on experience in a community involvement role (at least 2 years), communication skills and experience in facilitating group sessions. All staff had experience in delivering the programme prior to the commencement of the trial. An additional 2 days of training for Exeter-based facilitators (n=3) and 1 day for Birmingham-based facilitators (n=5) was provided by an expert in behaviour change (CG). The training focused on establishing the following competencies: Person-centred counselling (using reflective listening and other person-centred counselling techniques in a group setting); supporting behaviour change (including use of key motivational and self-regulatory behaviour change techniques listed above); providing accurate and guidance-adherent advice and information on diet; providing accurate and guidance-adherent advice and information on physical activity (including moderate and simple aerobic activity and muscle-strengthening exercise); supporting participants to address negative social influences; effective time management; and administration of the programme.

**Supplementary file 1 – References**

S1. National Institute for Health and Clinical Excellence. *Preventing type 2 diabetes: Risk identification and interventions for individuals at high risk* (PH38). London: NICE; 2012. <http://www.nice.org.uk/PH38> (Accessed 12/9/17).

S2. Greaves CJ, Sheppard KE, Abraham C, et al. Systematic review of reviews of intervention components associated with increased effectiveness in dietary and physical activity interventions. *BMC Public Health* 2011;11(119):1-12.

S3. Miller WR, Rollnick S. *Motivational interviewing: preparing people for change*. New York: The Guilford Press; 2002.

S4. Schwarzer R (1992). Self-efficacy in the adoption and maintenance of health behaviors: Theoretical approaches and a new model. In R. Schwarzer (Ed.), *Self-efficacy: Thought control of action*. Washington, DC: Hemisphere, pp. 217-242.

S5. Bandura A. *Social Foundations of Thought and Action*. Englewood Cliffs, NJ: Prentice-Hall; 1985.

S6. Carver CS, Scheier MF. Control theory: A useful conceptual framework for personality–social, clinical, and health psychology. *Psychol Bull* 1982;92(1):111-35.

S7. Deci EL, Ryan RM. The 'what' and 'why' of goal pursuits: Human needs and the self-determination of behavior. *Psychol Inq* 2000;11:227-68.

S8. Avenell A, Broom J, Brown TJ, et al. Systematic review of the long-term effects and economic consequences of treatments for obesity and implications for health improvement. *Health Technol Assess* 2004;8(21):1-194.

S9. Department of Health. *Start Active, Stay Active: A report on physical activity from the four home countries’ Chief Medical Officers*. London: Department of Health; 2011. [https://www.gov.uk/government/publications/start-active-stay-active-a-report-on-physical-activity-from-the-four-home-countries-chief-medical-officers](https://www.gov.uk/government/publications/start-active-stay-active-a-report-on-physical-activity-from-the-four-home-countries-chief-medical-officers%20Accessed%2024/1/14) (Accessed 28/11/17).

S10. Miller WR, Johnson WR. A natural language screening measure for motivation to change. *Addict Behav* 2008;33:1177-82.

S11. Abraham C, Michie S. A taxonomy of behavior change techniques used in interventions. [*Health Psychol*](http://www.ncbi.nlm.nih.gov/pubmed/18624603) 2008;27(3):379-87.
