# Supplementary material for: The community-based prevention of diabetes (ComPoD) study: a randomised, waiting list controlled trial of a voluntary sector-led diabetes prevention programme
Source: Int J Behav Nutr Phys Act. 2019 Nov 27;16:112. doi: 10.1186/s12966-019-0877-3 (PMC6880578; doi:10.1186/s12966-019-0877-3)
Supplement: Supplementary file 3 — Additional file 3. Supplementary tables. [file 12966_2019_877_MOESM3_ESM.docx]

**Supplementary Table 1. Characteristics of participants with and without primary outcome data at 6 and 12 months (n (%) unless indicated)**

|  |  | **6 months** | | | **12 months** | | |
| --- | --- | --- | --- | --- | --- | --- | --- |
|  |  | **Complete**  **n = 285** | **Missing**  **n = 29** | **p value for difference**^a^ | **Complete**  **n = 137**^b^ | **Missing**  **n = 20** | **p value for difference**^a^ |
| Male gender | | 124  (44) | 13  (45) | 0.891 | 59  (43) | 9  (45) | 0.870 |
| Mean (SD) age (years) | | 61.7  (9.6) | 58.2  (12.7) | 0.070 | 62.2  (9.7) | 56.2  (10.7) | 0.011 |
| White British ethnicity | | 211  (74) | 20  (69) | 0.555 | 108  (79) | 16  (80) | 0.905 |
| Currently in paid employment | | 104  (36) | 17  (59) | 0.020 | 43  (31) | 13  (65) | 0.003 |
| Education | School to <16y | 12  (4) | 2  (7) |  | 4  (3) | - |  |
|  | School to 16y | 96  (34) | 6  (21) |  | 44  (32) | 6  (30) |  |
|  | School to 18y | 17  (6) | 4  (14) |  | 8  (6) | 3  (15) |  |
|  | Further or higher education/training | 160  (56) | 17  (59) | 0.237 | 81  (59) | 11  (55) | 0.432 |
| Long term condition | | 211  (75) | 18  (62) | 0.147 | 100  (74) | 15  (75) | 0.930 |
| Disabled | | 35  (12) | 4  (14) | 0.816 | 14  (10) | 5  (25) | 0.058 |
| Smoking | | 32  (11) | 3  (10) | 0.881 | 18  (13) | 4  (20) | 0.417 |
| Median (IQR) IMD | | 19.7  (20.7) | 15.0  (25.9) | 0.788 | 19.5  (21.1) | 14.3  (24.3) | 0.829 |
| Mean (SD) weight (kg) | | 87.4  (15.4) | 86.3  (12.6) | 0.716 | 87.6  (17.2) | 88.6  (13.6) | 0.805 |
| Mean (SD) BMI | | 31.7  (4.6) | 32.0  (4.0) | 0.740 | 31.9  (4.7) | 31.7  (4.2) | 0.919 |

^a^Obtained from chi-square test for categorical outcomes, t-test where means or Mann-Whitney test where medians presented

^b^Includes 2 participants who provided self-reported weight data at 6 months not included in 12 month analyses

**Supplementary Table 2a. Birmingham participant characteristics and primary and secondary outcomes at baseline (n (%) unless indicated)**

|  | **Control**  n=71 unless indicated | **Intervention**  n=73 unless indicated | **Total sample**  **n=144 unless indicated** |
| --- | --- | --- | --- |
| **Stratification & minimisation variables** |  |  |  |
| Male gender | 28  (39) | 33  (45) | **61**  **(42)** |
| Age categories 18 - 54 years | 26 (37) | 25 (34) | **51 (35)** |
| 55 - 64 years | 20 (28) | 18 (25) | **38 (26)** |
| 65 - 75 years | 25 (35) | 30 (41) | **55 (38)** |
| Body mass index category 23 - 29.99 kg/m^2^ | 30 (42) | 32 (44) | **62 (43)** |
| 30 - 36.99 kg/m^2^ | 28 (39) | 29 (40) | **57 (40)** |
| 37 - 45 kg/m^2^ | 13 (18) | 12 (16) | **25 (17)** |
| **Socio-demographic & socio-economic characteristics** | |  |  |
| Mean (SD) age (years) | 58.78  (10.15) | 59.41  (10.76) | **59.10**  **(10.43)** |
| Ethnicity White British | 31 (44) | 42 (58) | **73 (51)*** |
| White other | 4 (6) | 1 (1) | **5 (3)** |
| Mixed | 1 (1) | 1 (1) | **2 (1)** |
| Asian | 23 (32) | 19 (26) | **42 (29)** |
| Black | 12 (17) | 10 (14) | **22 (15)** |
| Employment Retired | 26 (37) | 31 (42) | **57 (40)** |
| Employed/self-employed | 28 (39) | 24 (33) | **52 (37)** |
| Unemployed | 12 (17) | 12 (16) | **24 (17)** |
| Long-term sick/disabled | 2 (3) | 2 (3) | **4 (3)** |
| Carer | 1 (1) | 2 (3) | **3 (2)** |
| Other | 2 (3) | 2 (3) | **4 (3)** |
| Education Primary | 5 (7) | 2 (3) | **7 (5)** |
| Some secondary | 3 (4) | 2 (3) | **5 (3)** |
| Secondary to 16 years | 24 (34) | 23 (32) | **47 (33)** |
| Secondary to 18 years | 6 (8) | 5 (7) | **11 (8)** |
| Additional training | 22 (31) | 25 (34) | **47 (33)** |
| Undergraduate university | 8 (11) | 12 (16) | **20 (14)** |
| Postgraduate university | 3 (4) | 4 (5) | **7 (5)** |
| Median (IQR) Index of Multiple Deprivation score | 24.78  (23.48) | 24.78  (23.13) | **24.78**  **(22.98)** |
| Living in area with above average deprivation | 65  (92) | 65  (89) | **130**  **(90)** |
| **Clinical characteristics** |  |  |  |
| Any long term condition | 58  (82) | 58  (79) | **116**  **(81)** |
| Smoking | 8  (11) | 14  (19) | **22**  **(15)** |
| Disability | 8  (11) | 7  (10) | **15**  **(10)** |
| **Primary & secondary objective outcomes** |  |  |  |
| Mean (SD) weight (kg) | 86.38  (15.18) | 86.63  (19.50) | **86.50**  **(17.44)** |
| Mean (SD) body mass index (kg/m^2^) | 32.10  (4.95) | 31.81  (5.29) | **31.95**  **(5.11)** |
| Mean (SD) waist circumference (cm) | 107.41  (11.14) | 107.46  (13.54) | **107.44**  **(12.37)** |
| Mean (SD) HbA1c (mmol/mol) | 40.08  (2.79) | 40.04  (3.26) | **40.06**  **(3.03)** |
| Mean (SD) systolic blood pressure (mmHg) | 137.92  (18.45) | 131.74  (16.98) | **134.78**  **(17.93)** |
| Mean (SD) diastolic blood pressure (mmHg) | 80.79  (10.57) | 77.97  (11.83) | **79.36**  **(11.28)** |
| Mean (SD) total mins moderate-vigorous activity (MVPA) per week | 313.16  (167.57) | 347.06  (191.18) | **330.52**  **(180.12)** |
| Mean (SD) total mins MVPA in 10+ min bouts per week | 63.92^a^  (85.10) | 72.35^b^  (110.39) | **68.23^c^**  **(98.55)** |
| N (%) meeting recommendation for 150 mins MVPA in 10+ min bouts per week | 8^a^  (13) | 10^b^  (16) | 18^c^  (15) |
| **Self-reported outcomes** |  |  |  |
| Dietary behaviour fat subscale scores (0 to 4, lower better) | 1.98  (0.32) | 1.96  (0.33) | **1.97**  **(0.32)** |
| Dietary behaviour fibre subscale score (0 to 4, higher better) | 2.09  (0.38) | 2.09  (0.34) | **2.09**  **(0.36)** |
| Mean (SD) health rating (0-100) | 71.15  (16.30) | 70.02  (16.86) | **70.58**  **(16.54)** |
| Mean (SD) life satisfaction rating (0-10) | 7.30  (1.97) | 7.22  (2.15) | **7.26**  **(2.06)** |
| Mean (SD) mental well-being scores (0 – 35, higher better) | 24.92  (5.19) | 24.68  (4.49) | **24.80**  **(4.83)** |

*Compared to 53% for Birmingham population^55^

^a^n=60; ^b^n=63; ^c^n=123

**Supplementary Table 2b. Exeter participant characteristics and primary and secondary outcomes at baseline (n (%) unless indicated)**

|  | **Control**  n=86 unless indicated | **Intervention**  n=84 unless indicated | **Total sample**  **n=170 unless indicated** |
| --- | --- | --- | --- |
| **Stratification & minimisation variables** |  |  |  |
| Male gender | 41  (48) | 35  (42) | **76**  **(45)** |
| Age categories 18 - 54 years | 13 (15) | 14 (17) | **27 (16)** |
| 55 - 64 years | 23 (27) | 27 (32) | **50 (29)** |
| 65 - 75 years | 50 (58) | 43 (51) | **93 (55)** |
| Body mass index category 23 - 29.99 kg/m^2^ | 35 (41) | 32 (38) | **67 (39)** |
| 30 - 36.99 kg/m^2^ | 44 (51) | 43 (51) | **87 (51)** |
| 37 - 45 kg/m^2^ | 7 (8) | 9 (11) | **16 (9)** |
| **Socio-demographic & socio-economic characteristics** | |  |  |
| Mean (SD) age (years) | 63.35  (9.17) | 63.25  (8.96) | **63.30**  **(9.04)** |
| Ethnicity White British | 76 (88) | 82 (98) | **158 (93)*** |
| White other | 6 (7) | 1 (1) | **7 (4)** |
| Mixed | 1 (1) | - | **1 (1)** |
| Asian | 3 (3) | 1 (1) | **4 (2)** |
| Employment Retired | 47 (55) | 48 (57) | **95 (56)** |
| Employed/self-employed | 37 (43) | 32 (38) | **69 (40)** |
| Unemployed | - | 1 (1) | **1 (1)** |
| Long-term sick/disabled | - | 1 (1) | **1 (1)** |
| Carer | - | 2 (2) | **2 (1)** |
| Other | 2 (2) | - | **2 (1)** |
| Education Some secondary | 2 (2)^a^ | - | **2 (1)**^b^ |
| Secondary to 16 years | 28 (33)^a^ | 27 (32) | **55 (33)**^b^ |
| Secondary to 18 years | 4 (5)^a^ | 6 (7) | **10 (6)**^b^ |
| Additional training | 25 (29)^a^ | 34 (40) | **59 (35)**^b^ |
| Undergraduate uni | 16 (19)^a^ | 10 (12) | **26 (15)**^b^ |
| Postgraduate uni | 10 (12)^a^ | 7 (8) | **17 (10)**^b^ |
| Median (IQR) Index of Multiple Deprivation score | 13.13  (12.39) | 12.21  (10.23) | **12.54**  **(12.13)** |
| Living in area with above average deprivation | 30  (35) | 23  (27) | **52**  **(31)** |
| **Clinical characteristics** |  |  |  |
| Any long term condition | 56  (65) | 57^c^  (70) | **113**^d^  **(67)** |
| Smoking | 5  (6) | 8^e^  (10) | **13**^b^  **(8)** |
| Disability | 12  (14) | 12  (14) | **24**  **(14)** |
| **Primary & secondary objective outcomes** |  |  |  |
| Mean (SD) weight (kg) | 87.18  (11.74) | 88.72  (14.01) | **87.94**  **(12.90)** |
| Mean (SD) body mass index (kg/m^2^) | 31.12  (3.84) | 31.89  (4.08) | **31.50**  **(3.96)** |
| Mean (SD) waist circumference (cm) | 102.64  (10.99) | 102.59  (11.36) | **102.61**  **(11.14)** |
| Mean (SD) HbA1c (mmol/mol) | 39.37  (2.66) | 39.74  (2.88) | **39.55**  **(2.77)** |
| Mean (SD) systolic blood pressure (mmHg) | 137.30  (19.21) | 140.11  (16.33) | **138.69**  **(17.85)** |
| Mean (SD) diastolic blood pressure (mmHg) | 79.03  (10.61) | 79.83  (8.89) | **79.43**  **(9.78)** |
| Mean (SD) total mins moderate-vigorous activity (MVPA) per week | 323.08  (183.58) | 329.69  (188.33) | **326.49**  **(185.47)** |
| Mean (SD) total mins MVPA in 10+ min bouts per week | 81.26^f^  (104.93) | 86.19^g^  (122.03) | **83.80^h^**  **(113.74)** |
| N (%) meeting recommendation for 150 mins MVPA in 10+ min bouts per week | 14^f^  (19) | 15^g^  (19) | **29^h^**  **(19)** |
| **Self-reported outcomes** |  |  |  |
| Dietary behaviour fat subscale scores (0 to 3, lower better) | 1.92^e^  (0.43) | 1.93  (0.27) | **1.93**^i^  **(0.36)** |
| Dietary behaviour fibre subscale score (0 to 3, higher better) | 2.23^e^  (0.41) | 2.25  (0.39) | **2.24**  **(0.40)** |
| Mean (SD) health rating (0-100) | 78.53^j^  (17.65) | 75.21^e^  (22.14) | **76.85**^k^  **(20.05)** |
| Mean (SD) life satisfaction rating (0-10) | 7.54^j^  (1.83) | 8.02  (1.86) | **7.79**^l^  **(1.85)** |
| Mean (SD) mental well-being scores (0-35, higher better) | 24.85^e^  (5.04) | 24.94  (4.88) | **24.89**^i^  **(4.95)** |

*Compared to 88% for Exeter city, 95% for Devon^56^

^a^n=85; ^b^n=169; ^c^n=82; ^d^n=168; ^e^n=83; ^f^n=75; ^g^n=80; ^h^n=155; ^i^n=167; ^j^n=81; ^k^n=164; ^l^n=165

**Supplementary Table 3. Attendance patterns across prototype LWTC programme group sessions and follow-up contacts up to 6 months for those with complete group attendance data**

|  |  | **Group sessions attended** | | | | | | **Follow-up contacts^3^** | | |
| --- | --- | --- | --- | --- | --- | --- | --- | --- | --- | --- |
|  |  | **1** | **2** | **3** | **4** | **All 4** | **Mean (SD)** | **2m** | **3m** | **6m** |
| Bham^1^ | n with data | 62 | 62 | 62 | 62 | 62 | 62 | 60 | 60 | 47 |
|  | n (%) attending | 55 (89) | 47 (76) | 46  (74) | 45 (73) | 35  (56) | 3.11  (1.23) | 41 (68) | 43 (72) | 33 (70) |
| Exeter^2^ | n with data | 67 | 67 | 67 | 67 | 67 | 67 | 49 | 31 | 12 |
|  | n (%) attending | 64 (96) | 61 (91) | 60  (90) | 60 (90) | 53  (79) | 3.66  (0.83) | 34  (70) | 20  (65) | 10 (83) |
| **TOTAL** | **Total n** | **129** | **129** | **129** | **129** | **129** | 129 | **109** | **91** | **59** |
|  | **n (%) attending** | **119 (92)** | **108 (84)** | **106**  **(82)** | **105 (81)** | **88**  **(68)** | **3.40**  **(1.07)** | **75 (69)** | **63 (69)** | **43 (73)** |

^1^In Birmingham, all 2- and 3-month follow-ups for which data were available were conducted via phone, and all 6-month contacts were conducted in a group

^2^In Exeter, at 2 months all but one participant (97%) for whom data were available received follow up in a group, and at 3 and 6 months all of the limited number of participants for whom data were available were followed up in a group.

^3^Data on contacts beyond 6 months were available for only six (4%) participants.

**Supplementary Table 4. Characteristics of participants attending and not attending all 4 group sessions (n (%) unless indicated)**

|  |  | Not attending all 4 group sessions  n = 41 | Attending all 4 group sessions  n = 88 | p value for difference^a^ |
| --- | --- | --- | --- | --- |
| Male gender |  | 16  (39) | 41  (47) | 0.420 |
| Mean (SD) age (years) | | 57.9  (10.6) | 64.6  (8.1) | 0.001 |
| White British ethnicity | | 27  (66) | 76  (86) | 0.007 |
| Employment | Employed | 17  (41) | 28  (32) | 0.284 |
| Education | School to <16y | 2  (5) | 1  (1) |  |
|  | School to 16y | 10  (24) | 32  (36) | 0.020 |
|  | School to 18y | - | 9  (10) |  |
|  | Further or higher education/training | 29  (71) | 46  (52) |  |
| Long term condition | | 25  (61) | 66  (76) | 0.083 |
| Disabled |  | 5  (12) | 11  (13) | 0.961 |
| Smoking |  | 8  (20) | 6  (7) | 0.033 |
| Median (IQR) IMD | | 21.4  (21.7) | 18.6  (13.8) | 0.025 |
| Mean (SD) weight (kg) | | 87.6  (20.4) | 88.1  (15.6) | 0.873 |
| Mean (SD) BMI (kg/m^2^) | | 31.6  (4.8) | 32.1  (4.7) | 0.626 |

^a^Obtained from chi-square/Fishers exact test for categorical outcomes, t-test where means or Mann-Whitney test where medians presented

**Supplementary Table 5. Secondary Intention-to-treat analyses for weight loss at 6 months using different methods to impute missing primary outcome data**

|  | **6 month follow-up** | |  |  |
| --- | --- | --- | --- | --- |
| **Method of imputation** | Control  n=157  unless indicated | Intervention  n=157  unless indicated | Between group difference*  Mean (95% CI) | p-value |
| Self-reported weight | -0.08^a^  (3.03) | -1.88^b^  (4.43) | -1.73  (-2.58 to -0.87) | <0.001 |
| Multiple imputation | N/A | N/A | -1.73  (-2.61 to -0.85) | <0.001 |
| Baseline observation carried forward | -0.09  (2.90) | -1.72  (4.29) | -1.55  (-2.34 to -0.75) | <0.001 |
| Worst case scenario (control: mean weight loss in control completers; intervention: no change) | -0.10  (2.90) | -1.72  (4.29) | -1.54  (-2.33 to -0.75) | <0.001 |
| Best case scenario (control: no change; intervention: weight loss in intervention completers) | -0.09  (2.90) | -1.91  (4.25) | -1.74  (-2.53 to -0.95) | <0.001 |

^a^n=146; ^b^n=147

**Supplementary Table 6. Intention-to-treat completers analysis of primary and secondary outcomes at 6 months with additional adjustment for baseline differences in ethnicity**

|  | **Waiting list control group**  N=144, mean (SD) unless indicated | | **Intervention group**  N=141, mean (SD) unless indicated | | Between group difference*  Mean (95% CI) or odds ratio | p-value |
| --- | --- | --- | --- | --- | --- | --- |
|  | Baseline | 6-months | Baseline | 6-months | (OR) (95% CI) where indicated |  |
| **Primary outcome** |  |  |  |  |  |  |
| Weight loss (kg) | n/a | -0.10  (3.03) | n/a | -1.91  (4.49) | -1.87  (-2.74 to -1.00) | <0.001 |
| **Secondary weight-related outcomes** |  |  |  |  |  |  |
| N (%) >3% weight loss | n/a | 23  (16) | n/a | 46  (33) | OR = 2.80  (1.54 to 5.10) | 0.001 |
| N (%) >5% weight loss | n/a | 12  (8) | n/a | 29  (21) | OR = 3.19  (1.49 to 6.83) | 0.004 |
| BMI (kg/m^2^) | 31.56  (4.39) | 31.58  (4.60) | 31.85  (4.66) | 31.07  (4.53) | -0.69  (-1.00 to -0.37) | <0.001 |
| Waist circumference (cm) | 104.80  (11.28) | 104.11  (11.48) | 104.85  (12.62) | 102.36  (12.50) | -1.63  (-2.63 to -0.64) | 0.001 |
| **Other secondary objective outcomes** |  |  |  |  |  |  |
| HbA1c (mmol/mol) | 39.69  (2.73) | 40.18^a^  (3.22) | 39.88  (3.06) | 39.71  (5.70) | -0.72  (-1.70 to 0.26) | 0.148 |
| Blood pressure systolic (mmHg) | 137.58  (18.82) | 138.44  (17.20) | 136.22  (17.10) | 136.91  (17.48) | -0.53  (-3.76 to 2.70) | 0.747 |
| Blood pressure diastolic (mmHg) | 79.83  (10.60) | 79.85  (9.33) | 78.97  (10.37) | 78.21  (9.77) | -0.84  (-2.68 to 1.00) | 0.368 |

*Mean differences, odds ratios and CIs adjusted for baseline outcome value, site, gender, age, baseline BMI category and ethnicity (White British versus other)

^a^n=142, 2 participants missing HbA1c due to machine malfunction
